# Rubella Vaccination Coverage Among Women of Childbearing Age in Vietnam

**DOI:** 10.3390/ijerph16101741

**Published:** 2019-05-16

**Authors:** Toan Thanh Thi Do, Anh Ngoc Nguyen, Xuan Thanh Thi Le, Ann Pongsakul, Quang Nhat Nguyen, Thanh Van Nguyen, Thang Huu Nguyen, Tri Minh Do, Huong Thi Le, Huong Lan Thi Nguyen, Nu Thi Truong, Chi Linh Hoang, Giang Thu Vu, Tung Thanh Tran, Tung Hoang Tran, Bach Xuan Tran, Carl A. Latkin, Cyrus SH Ho, Roger CM Ho

**Affiliations:** 1 Institute for Preventive Medicine and Public Health, Hanoi Medical University, Hanoi 100000, Vietnam; dothithanhtoan@hmu.edu.vn (T.T.T.D.); ngocanh0407hmu@gmail.com (A.N.N.); nguyenvanthanh.lhp@gmail.com (T.V.N.); thangtcyt@gmail.com (T.H.N.); dominhtri37@yahoo.com.vn (T.M.D.); lethihuong@hmu.edu.vn (H.T.L.); bach.ipmph@gmail.com (B.X.T.); 2 West Virginia School of Osteopathic Medicine, Lewisburg, WV 24901, USA; ppongsakul@osteo.wvsom.edu; 3 Université Claude Bernard Lyon 1, 69100 Villeurbanne, France; quang.n.nguyen@alumni.duke.edu; 4 Institute for Global Health Innovations, Duy Tan University, Da Nang 55000, Vietnam; huong.ighi@gmail.com; 5 Center of Excellence in Behavior Medicine, Nguyen Tat Thanh University, Ho Chi Minh 70000, Vietnam; nu.coentt@gmail.com (N.T.T.); chi.coentt@gmail.com (C.L.H.); 6 Center of Excellence in Evidence-Based Medicine, Nguyen Tat Thanh University, Ho Chi Minh 70000, Vietnam; giang.coentt@gmail.com (G.T.V.); tung.coentt@gmail.com (T.T.T.); 7 Department of Lower Limb Surgery, Vietnam-Germany Hospital, Hanoi 100000, Vietnam; tranhoangtung.vd@gmail.com; 8 Johns Hopkins Bloomberg School of Public Health, Baltimore, MD 21205, USA; carl.latkin@jhu.edu; 9 Department of Psychological Medicine, National University Hospital, Singapore 119074, Singapore; cyrushosh@gmail.com; 10 Department of Psychological Medicine, Yong Loo Lin School of Medicine, National University of Singapore, Singapore 119077, Singapore; pcmrhcm@nus.edu.sg

**Keywords:** rubella vaccination, CRS, pregnant women, women of childbearing age, Vietnam

## Abstract

Despite the availability of effective and safe rubella vaccines for women of childbearing age, prevention and control of congenital rubella syndrome in children remains challenging in Vietnam. In order to examine this issue, we conducted a cross-sectional study, examining the current coverage of rubella vaccination before pregnancy among 807 pregnant women and women with children under 12 months of age in urban and rural districts, Dong Da and Ba Vi, in Hanoi, Vietnam. In this population, we observed an alarming non-compliance rate with rubella vaccination before pregnancy in both localities. Among the 82.0% of participants who remained unvaccinated against this contagious viral infection, 95.8% of them were in Ba Vi district, compared to 68.0% in Dong Da district (*p* < 0.001). Besides the differences in age, number of children, education levels, primary occupations and monthly incomes among the participants between the two districts, other reasons for noncompliance with rubella vaccination includeddisinterest in rubella vaccination, the high cost and long distance to vaccination sites as well as unawareness of vaccination locations. In addition to addressing the unique socio-economicchallenges behind one’s accessibility to vaccination services in urban and rural areas, our study supports a continued effort in ensuring proper access to and education about pre-pregnancy vaccines and vaccination among women of childbearing age in order to achieve and sustain sufficient immunization coverage of rubella and other vaccine-preventable diseases in both settings.

## 1. Introduction

Rubella is a highly contagious viral disease, primarily affecting susceptible children and women of childbearing age [1]. For pregnant women in their first trimester of pregnancy, if infected, rubella may also cause fetal infection, resulting in abnormal fetal growth and even miscarriage. Moreover, rubella infection in early pregnancy may lead to congenital rubella syndrome (CRS) [1]. Babies born to rubella-infected pregnant mothers are at risk of developing numerous severe birth defects, such as deafness, cataracts, heart defects and developmental delays, due to CRS [1]. A number of child deaths have in fact been recorded as the result of CRS [2,3,4]. Although there is no cure orspecific treatment for rubella, the rubella vaccination, either as single-dose or combined vaccines (measles-rubella (MR) or measles-mumps-rubella (MMR)), can prevent the infection and its serious health complications [2]. It is worth noting that since rubella vaccines are not recommended for pregnant women, vaccination against rubella among women of childbearing age is important to prevent rubella infection during pregnancy as well as CRS in children.

Globally, as of 2014, 140 out of 194 countries have rubella coverage, compared to 99 countries with rubella vaccine coverage in 2000 [5]. With the introduction of the rubella-containing vaccine (RCV), the coverage of the first RCV dose was estimated to increase to 15.0% in 2016 from 3.0% in 2000in the Southeast Asia region [6]. It has also been estimated that over 80 million persons in the Southeast Asian countries, where supplemental immunization activities were conducted, got vaccinated during 2000–2016 [6]. In the same period, a decrease in rubella cases was also observed in some of these countries [6]. Nevertheless, despite the availability of a safe and effective rubella vaccine, which could achieve over 95.0% long-lasting immunity after just one dose, data from the Centers for Disease Control and Prevention in 2014 still reported a total of 33,068 cases of rubella in 161 countries [7,8,9]. The prevalence of rubella infection in the Southeast Asia region, in particular, is ranked second in the world with 9263 cases, following the Western Pacific region, which differs significantly from the Americas region in which rubella vaccine coverage was established in all 35 countries and there were only 4 reported cases of rubella infection in 2014 [5]. In Vietnam, a significant unmet need to vaccinate women of childbearing age to prevent and reduce the high burden of rubella infections during pregnancy, resulting in miscarriages as well as CRS, has been reported [9,10,11]. Yet, a pandemic of rubella occurred in 2011, causing 2000 pregnant women to become infected with rubella [9]. Among which, only about 1000 rubella-infected women visited the Center for Prenatal Diagnosis of the National Hospital of Obstetrics and Gynecology for consultation, and nearly 100 infants were infected with CRS [9].

In response, Vietnam launched its largest nation-wide campaign, the National Expanded Immunization Program (NEIP), to increase rubella vaccination in children ages 1–14 years in 2014 [5]. In Vietnam, rubella vaccines are currently administered in a variety of settings, with most being administered at public Commune Health Stations, followed by the Centers for Vaccination Services. Commune Health Stations provide MR vaccines as part of pregnancy care, and are free of charge thanks to the NEIP. They also offer primary health care services, including reproductive and maternal care. On the other hand, the Centers for Vaccination Services administer the MMR vaccine as part of pre-pregnancy and pregnancy care. Although these centers offer faster vaccination services, they do not provide other health services aside from vaccinations, and the vaccines need to be paid for by the patients [12]. It is worth noting, however, that women of childbearing age were not covered under the NEIP, leaving a high risk of CRS among children born to unvaccinated women of childbearing age [13].

Besides the differences in vaccination services and coverage among different health clinics, the disparity in maternal and childhood vaccination between urban and rural areasmight also contribute to the persistence of rubella infections in Vietnam. In other countries, geographicalisolation, and the associated socio-economic differences, havein fact been shown to influence vaccination coverage [14,15,16]. However, such knowledge in the context of Vietnam remains largely unexplored. Interestingly, although the childhood immunization rate in the rural areas in Vietnam has generally been lower than that in the urban areas, both the urban and rural districts of Dong Da and Ba Vi, respectively, were among the top five districts with the highest number of rubella cases from the 233 cases reported in Hanoi in 2011 [13]. In particular, Dong Da district reported 22 cases in 11 wards, and Ba Vi district reported 20 cases in nine communes [13]. Regarding the vaccination services in these localities, Dong Da is an urban district in the center of Hanoi in which maternal and infant healthcare services, including MMR vaccines, have been available since 2001 at various health service facilities. In contrast, Ba Vi district is primarily an agricultural commune with 84% of people working in farming. In Ba Vi district, there has not been a strong implementation of such vaccination services, resulting in people having to travel outside of the district to get vaccinated. Moreover, the district has experienced a severe lack of facilities, such as injection rooms and health workers, until around the end of 2016 when it started to develop a plan to provide service vaccines [13]. It is thus likely that the differences in vaccination services and infrastructure could account for the persistently high prevalence of rubella in these districts. Nonetheless, the extent to which these differences and other factors, such as socio-economic status, could contribute to the insufficient control of rubella infections in urban and rural areas remains poorly investigated.

In this study, we assessed the current rubella vaccination coverage before pregnancy and evaluated the potential reasons for rubella vaccination non-compliance among pregnant women and those of childbearing age when rubella vaccination is highly recommended in the Dong Da and Ba Vi districts in Hanoi, Vietnam. Such knowledge might provide important insights into developing future vaccination strategies to address the disparity in vaccination services and coverage against rubella and other vaccine-preventable diseases in urban and local areas.

## 2. Materials and Methods

### 2.1. Study Setting, Sample Size and Sampling Method

A cross-sectional study was conducted from February to June 2016. The sample for the survey was selected from one urban and one suburban districts in Hanoi City. Dong Da district, which has 21 wards, is located in the center of Hanoi and is the most populated district with anaverage population of352,000 people. However about 10.0% of peopleare temporary migrants doing trade or seasonal work. Ba Vi district, with one town and 30 communes, is a suburban district located in northwestern Hanoi with anaverage population of278,000 people. In each district, twowards/communes were selected randomly. Every women who (1) had never been infected with rubella, (2) waseither pregnant or had children who were less than 12 months of age and (3) agreed to participate in the study, had been selected from the report of health workers for each ward/commune. Health workers of each commune health center hadmade a list of all eligible study participants following the study criteria. Then, convenient sampling was applied to select all eligible study participants who agreed to participate the study. A total of 807 eligible women had been recruited. Of these, 400 lived in Dong Da district, and 407 lived in Ba Vi district. The investigation was then conducted among the women selected by the population group.

### 2.2. Measures and Instruments

A pre-designed questionnaire in Vietnamese was used to obtain participants information regarding their age, education level, current primary occupation, average monthly income per capita and current number of children. This questionnaire was pretested in five pregnant women with children less than one year of age in Hanoi, Vietnam, prior to its utilization in this study, with each item beingmeasured havingan Alpha reliability greater than 0.6. In this study, sufficient vaccination against rubella before pregnancy is defined as receiving at least one dose of rubella vaccine within at least three months prior to pregnancy, according to current recommendations [4]. Of note, the reasons behind non-compliance with rubella vaccination before pregnancy were qualitatively determined by the participants.

### 2.3. Statistical Analysis

Data wereanalyzed using descriptive statistics, and the chi-squared test was applied to comparethe demographic characteristics and prevalence of rubella vaccinationinparticipants between the two study sites. When the frequency was smaller than 5, we applied Fisher’s exact test.

Univariate analysis was applied to calculate the odds ratio (OR) with 95% confidence intervals (95%CIs) for the dependent variable (utilization of Rubella vaccine) and each independent variable (characteristics of women: age, living area, occupation, educational level and number of children; knowledge ofrubella disease and rubella vaccine and attitude toward rubella vaccination). All variables found to have a statistically significant association (*p*-value < 0.05) with rubella vaccination uptake in the univariate analysis were included in the multivariable stepwise logistic regression models. Significant interaction effects were also tested to identify the specific differences between some covariates (knowledge ofrubella associated with knowledge ofrubella vaccine). If there was significant interaction, it was put into the model. Adjusted OR (adj-OR) with 95%CIs was reported in the final model. The final model was the best fit model with the lowest Akaike’s Information Criterion (AIC). Log-likelihood is ameasure of model fit.

### 2.4. Ethical Considerations

This study was approved by the Ethics Committee of Hanoi Medical University (Code number:184/HMU-IRB dated 14 November 2015).Following the one-on-one explanation of the study by trained healthcare workers at the Hanoi Medical University, every participant gave verbal informed consents prior to his/her participation in the studies, acknowledging a full understanding of the study’s purpose, his/her rights to withdraw from the study at any time and the protection of confidentiality to the participant.

## 3. Results

### 3.1. Demographic Characteristics of Participants

Of 807 participants, 47.1% of the participants were between 25 and 30 years old, and 35.1% of them were above 30 years old (Table 1). This age distribution was similar in Dong Da district; however, there were 27.3% of participants below the age of 25 in Ba Vi district compared to 8% in Dong Da district. Of note, the average childbearing age in Vietnam is 24.6 years. In the present study population, 80.5% of the participants had obtained at least a high school diploma from which 69.6% of them lived in Ba Vi district, and 89.5%of them lived in Dong Da district. Regarding their current primary occupation, most of the participants (41.4%) were housewives/farmers, as similarly observed in Ba Vi district (72%). In Dong Da district, on the other hand, most were either public servants (47%), e.g., those who worked in state administrative agencies, or businesspeople/traders (39.8%). Financially, most participants (88.6%) reported making above five million VND a month on average, of which, 78.1% of the participants lived in Ba Vi district, and 99.2% of them lived in Dong Da district. Lastly, almost all of the participants in both districts (99.0%) had more than one child. Between the two districts, significant differences were observed in the participants’ age groups, education levels, occupations and the average monthly income per capita (*p* < 0.01).

### 3.2. Coverage of Rubella Vaccination Before Pregnancy

**Figure 1:**
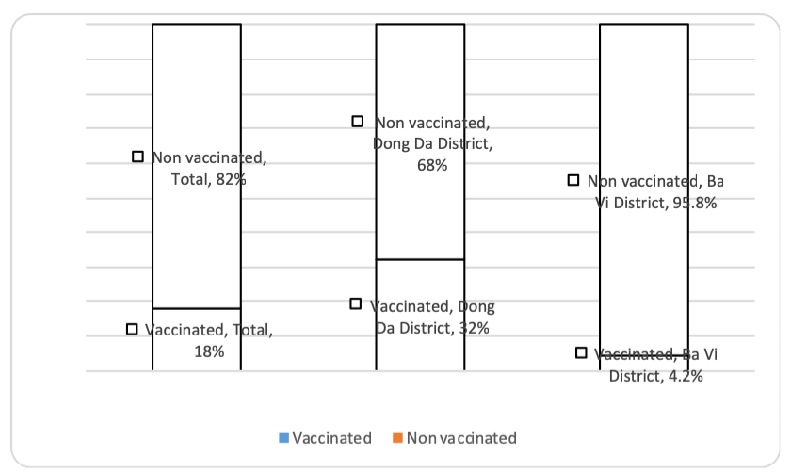
Rubella vaccination coverage before pregnancy.

In the present study, 82.0% of participants reported not having received sufficient rubella vaccination prior to pregnancy (Figure 1). Particularly, this high non-compliance rate was more pronounced in Ba Vi district than in Dong Da district (95.8% vs. 68%, *p* < 0.001). Among those who did receive rubella vaccines, the majority of them (87.5%) had received a single dose of rubella vaccine, and only 10 participants (12.5%) reported having received two doses. Regarding the type of rubella vaccines, 59.3% of participants received the combined MMR vaccines, and 22.7% of them received rubella monovalent vaccines.

### 3.3. Utilization of Vaccination Sites

Of 145 participants who received rubella vaccines, 17 participants (of 407 participants who lived in Ba Vi district) went to vaccination sites in Ba Vi district, and 128 participants (of 400 participants who lived in Dong Da district) went to vaccination sites in Dong Da district (Table 2). The majority of participants (44.1%) received rubella vaccines at the Centers for Vaccination Services, which was similarly observed in 46% of participants in Dong Da district. However, in Ba Vi district, only 29.4% of participants went to Centers for Vaccination Services, whereas 41.1% of them went to Commune Health Stations. No significant difference was observed in the utilization of vaccination sites between the two districts (*p* > 0.05).

### 3.4. Reasons Behind Non-Compliance withRubellaVaccination Before Pregnancy

Among the 662 participants who did not receive rubella vaccines before pregnancy, the majority of them reported being unaware of either the vaccination sites (19.7%) or of rubella vaccines (14.6%), and were not interested in rubella vaccination (19.5%) (Table 3). Of note, 7.3% of participants—belonging to the Others category—said they refused rubella vaccination due to “fear of vaccination,” “advanced maternal age” and “allergy to vaccine components.” Between the two study sites, there were more participants who claimed disinterest in rubella vaccination in Dong Da district than in Ba Vi district (22.3% vs. 16.7%, *p* < 0.001). Conversely, there were more participants in Ba Vi district than in Dong Da district who claimed the reasons behind their non-compliance with rubella vaccination included their unawareness of vaccination sites, high vaccination cost, long distance from their residence to vaccination sites or being too busy to get to vaccination sites (*p* < 0.001, and *p* < 0.01).

### 3.5. Factors Associated with Rubella Vaccination Uptake Before Pregnancy

Factors associated with the uptake of the rubella vaccine before pregnancy are presented in Table 4. In the multivariate analysis, the uptake of rubella vaccinations was strongly associated with women living in the urbanDong Da district (adj OR 8.2; 95%CI: 4.43–15.26) and having an age over 25 (adj OR 2.88; 95%CI: 1.26–6.60). Related to the awareness of women on needing to be vaccinated, those aware of the necessity to uptake vaccine is 4.97 times compared to the unaware group (adj OR 4.97; 95%CI: 2.06–11.99). On the contrary, women who have more than two children were less likely to have therubella vaccine before pregnancy than women who have lower than two (adj-OR 0.6; 95%CI: 0.37–0.97).

## 4. Discussion

Despite the tremendous national effort to increase childhood vaccination compliance, rubella outbreaks still occur in Vietnam and elsewhere, posing a great risk for both maternal and child health [11,17]. In this study, we observed an alarmingly high rate of non-compliance with rubella vaccination before pregnancy among pregnant women and those with children at one year of age in both urban and rural districts, Dong Da and Ba Vi, respectively. This lack of vaccination compliance was particularly observed in Ba Vi district, with 95.8% of the participants remaining unvaccinated against rubella. Between the two districts, we observed differences in the participants’ age, education level, primary occupation and average monthly income, but not in their utilization of vaccination sites. Further investigations revealed several reasons for non-compliance, including disinterest in rubella vaccination among participants in the urban district, and high vaccination cost, long distance to and lack of awareness of vaccination sites among participants in the rural district.

Although both urban and rural districts in our study reported a high non-compliance rate of vaccination against rubella, the significantly lower compliance in the rural district suggests a need for improvement in the vaccination strategy and outreach in this setting, such as ones afforded through the NEIP. For example, as similarly observed in our study, besides the exclusion of women of childbearing age, one challenge that the NEIP faces is reaching the target population with less access to basic healthcare and vaccination services, such as those having lower income and education levels, as well as those who belong to minority ethnic groups and live in rural areas [18]. In fact, even though the relationship between parental education about vaccines and vaccination coverage was not directly explored, parental education, including paternal education status, has been associated with immunization coverage [16,19]. Enhanced education about vaccines and vaccination, targeting adolescent girls before their childbearing ageor during pre-pregnancy and pregnancy counseling, might therefore improve vaccination coverage and reduce serious disease complications [10]. Additionally, as seen in our study, logistics challenges, such as being unaware of where to get vaccinated, long distances to vaccination sites and concerns about the associated cost and time commitment were indeed more common among participants in the rural district. Thus, although a difference in the utilization of vaccination sites between the two districts was not detected in our study, the lower rubella vaccination rate in the rural area in our study might speak to the potential disparity in healthcare infrastructure and vaccination services in rural and urban areas. For example, commune health stations in the rural district might be more convenient for people to receive vaccine services by providing both primary health care services and vaccination services. However, such convenience to access free-of-charge vaccination services appeared to be insufficient to fully address the high demand for pre-pregnancy vaccines and the shortage of vaccine supply [12,18]. On the other hand, the Centers for Vaccination Services in urban districts, which only specialize in vaccine services, including pre-pregnancy vaccines, are less likely to get crowded and can offer a faster option for busy city workers, but they do not accept any form of insurance [12,20]. In addition, we found a higher association with rubella vaccination uptake among women over age 25, women who have less than two children andwomen who are unaware of the advantage of rubella vaccination. Therefore, future vaccination strategies should be tailored to address the specific needs and challenges among populations with different socioeconomic statuses, which have been shown to influence vaccination uptake in urban and rural settings [16,21].

In this study, participants in the rural district expressed more of a concern for accessibility to affordable vaccines than participants in the urban district. Moreover, we found a small, yet considerable, proportion of participants who reported doubts and concerns about the well-demonstrated safety and efficacy of rubella vaccines, which could be indicative of the persisting influence of the anti-vaccination movements [7,8,22]. It is important to note, however, that such movement by vocal anti-vaccine activists, who inaccurately disseminate information about vaccines and vaccinations, could negatively misinform people of various socioeconomic levels across urban and rural communities [23].Given that vaccination remains the most effective strategy to prevent a rubella outbreak and its potentially deleterious health complications in children and young adults, increased education and awareness of the rubella infection risks and the rubella vaccine efficacy among parents in both urban and rural areas are warranted to address vaccine hesitancy and potentially increase vaccination coverage [22].

Several limitations exist in our study. First, besides our inability to draw a causal relationship, or any association between the reported reasons of vaccination non-compliance and the observed vaccination coverage, self-reporting bias in participants’ report of their reasons for not getting the rubella vaccine should be taken into account when interpreting our results. Second, although the Ba Vi and Dong Da districts serve as good proxies for assessing rubella vaccination coverage given their high rates of rubella cases, they might not represent all features from other regions that could further explain the discrepancy in vaccination coverage between the rural and urban settings in Vietnam. Thirdly, the convenient sampling applied to gather study participants mighthave caused selection bias due to some eligible women beingabsent during the time of the study. Lastly, we only examined a woman of childbearing age in this study. Even though such a population is the prime target of rubella vaccination against CRS in children, the husbands’ attitude and knowledge about vaccines and vaccination might also contribute to their wives’ decision to get vaccinated. Future study should, therefore, consider the whole family in examining one’s reasons for not being compliant with vaccination.

## 5. Conclusions

Despite the availability of effective and safe rubella vaccines for women of childbearing age, rubella infection and congenital rubella syndrome in children remain a public health risk in Vietnam. Here we reported a high non-compliance rate with rubella vaccination before pregnancy in both urban and rural districts in Vietnam. In addition to several socioeconomic challenges in the rural district, other reasons for such non-compliance, including disinterest in rubella vaccination and logistics issues inaccessing affordable vaccines, appeared to differ between participants living in the rural and urban districts. Given the importance of pre-pregnancy rubella vaccination in maternal and child health, continued effort in ensuring proper access to, availability of and education about rubella vaccines and vaccination services among families, especially those in rural areas, might help to improve and sustain the sufficient immunization coverage of rubella and other vaccine-preventable diseases.

## Figures and Tables

**Table 1:** Demographic characteristics of participants.

Characteristics		Ba Vi District (*n* = 407)		Dong Da District (*n* = 400)		Total (*n* = 807)	
		*n*	%	*n*	%	*n*	%
**Age group** ^1^	Under 25	111	27.3	33	8.3	144	17.8
	25–30	193	47.4	187	46.7	380	47.1
	Above 30	103	25.3	180	45.0	283	35.1
**Education level** ^1^	High school or above	283	69.6	358	89.5	650	80.5
	Below high school	124	30.4	42	10.5	157	19.5
**Primary occupation** ^1^	Housewife/Farming	293	72.0	41	10.2	334	41.4
	Public servant	34	8.3	188	47.0	222	27.5
	Manual Labor Worker	45	11.0	12	3.0	57	7.1
	Business person/Trader	35	8.7	159	39.8	194	24
**Average monthly income per capita (VND)** ^1^	Under 3 million	11	2.7	1	0.3	12	1.5
	3–5 million	78	19.2	2	0.5	80	9.9
	Above 5 million	318	78.1	397	99.2	715	88.6
**Current number of children**	First time being pregnant	6	1.5	2	0.5	8	1.0
	More than one child	401	98.5	398	99.5	799	99.0

^1^*p* < 0.001.

**Table 2:** Utilization of vaccination sites.

Vaccination Sites	Ba Vi District (*n* = 17)		Dong Da District (*n* = 128)		Total (*n* = 145)	
	*n*	%	*n*	%	*n*	%
**Commune health stations**	7	41.1	19	14.8	26	17.9
**Provincial/District hospitals**	4	28.6	2	1.6	6	4.1
**District health centers**	1	5.9	3	2.3	4	2.8
**Provincial preventive medicine centers**	2	11.8	30	23.4	32	22
**Private clinics**	0	0	4	3.1	4	2.8
**Private/Foreign hospitals**	1	5.9	7	5.5	8	5.5
**Central hospitals**	1	5.9	7	5.5	8	5.5
**Centers for vaccination services**	5	29.4	59	46	64	44.1
**Others**	0	0	4	3.1	4	2.8

**Table 3:** Reasons behind non-compliancewith rubella vaccination before pregnancy.

Reasons Behindrubella Vaccination Non-Compliance before Pregnancy	Ba Vi District (*n* = 390)		Dong Da District (*n* = 272)		Total (*n* = 662)	
	*n*	%	*n*	%	*n*	%
**Not interested in rubella vaccination** **(*p* < 0.001)**	68	16.7	89	22.3	157	19.5
**Unaware of vaccination sites (*p* < 0.001)**	151	37.1	8	2.0	159	19.7
**High cost (*p* < 0.001)**	49	12.0	17	4.3	66	8.2
**Long distance (*p* < 0.001)**	35	8.6	25	6.3	60	7.4
**Unaware of vaccines against rubella**	23	5.7	95	23.8	118	14.6
**Busy/having no time (*p* < 0.001)**	62	15.2	38	9.5	100	12.4
**Others**	4	1.0	55	13.8	59	7.3

**Table 4:** Multivariable logistic regression analysis of factors associated with the utilization of the rubella vaccine.

Factors	Options	Crude OR (95%CIs)	Adj OR ^a^ (95%CIs)
Age group	<25	Referent	Referent
	≥25	4.89 (2.30–10.4)	2.88 (1.26–6.60)
Living area	Dong Da	10.79 (6.11–19.05)	8.2 (4.43–15.26)
	Ba Vi	Referent	Referent
Number of children	≤1 child	Referent	Referent
	>2 children	0.59 (0.4–0.87)	0.6 (0.37–0.97)
Need to have vaccination	Not necessary	Referent	Referent
	Necessary	4.93 (2.1–2.58)	4.97 (2.06–11.99)
Knowledge on rubella disease	Not good	Referent	Referent
	Good	1.77 (1.21–2.58)	1.12 (0.64–1.96)
Knowledge on rubella vaccine	Not good	Referent	Referent
	Good	2.67 (1.73–4.12)	1.4 (0.72–2.64)

^a^: Adjusted for occupation and education. Constant of model β_0_: 0.057; Pseudo R^2^ of logistic regression mode = 0.254; AIC = 557.94; OR: odds ration; CI: confidence interval.
